# Why antidiabetic drugs are potentially neuroprotective during the Sars-CoV-2 pandemic: The focus on astroglial UPR and calcium-binding proteins

**DOI:** 10.3389/fncel.2022.905218

**Published:** 2022-07-29

**Authors:** Carlos-Alberto Gonçalves, Patrícia Sesterheim, Krista M. Wartchow, Larissa Daniele Bobermin, Guilhian Leipnitz, André Quincozes-Santos

**Affiliations:** ^1^Programa de Pós-Graduação em Ciências Biológicas: Bioquímica, Instituto de Ciências Básicas da Saúde, Universidade Federal do Rio Grande do Sul, Porto Alegre, RS, Brazil; ^2^Programa de Pós-Graduação em Neurociências, Instituto de Ciências Básicas da Saúde, Universidade Federal do Rio Grande do Sul, Porto Alegre, RS, Brazil

**Keywords:** astrocyte, calcium-binding proteins, COVID-19, diabetes mellitus, neuroprotection, UPR

## Abstract

We are living in a terrifying pandemic caused by Sars-CoV-2, in which patients with diabetes mellitus have, from the beginning, been identified as having a high risk of hospitalization and mortality. This viral disease is not limited to the respiratory system, but also affects, among other organs, the central nervous system. Furthermore, we already know that individuals with diabetes mellitus exhibit signs of astrocyte dysfunction and are more likely to develop cognitive deficits and even dementia. It is now being realized that COVID-19 incurs long-term effects and that those infected can develop several neurological and psychiatric manifestations. As this virus seriously compromises cell metabolism by triggering several mechanisms leading to the unfolded protein response (UPR), which involves endoplasmic reticulum Ca^2+^ depletion, we review here the basis involved in this response that are intimately associated with the development of neurodegenerative diseases. The discussion aims to highlight two aspects—the role of calcium-binding proteins and the role of astrocytes, glial cells that integrate energy metabolism with neurotransmission and with neuroinflammation. Among the proteins discussed are calpain, calcineurin, and sorcin. These proteins are emphasized as markers of the UPR and are potential therapeutic targets. Finally, we discuss the role of drugs widely prescribed to patients with diabetes mellitus, such as statins, metformin, and calcium channel blockers. The review assesses potential neuroprotection mechanisms, focusing on the UPR and the restoration of reticular Ca^2+^ homeostasis, based on both clinical and experimental data.

## Introduction

We are still experiencing a terrible pandemic caused by severe acute respiratory syndrome coronavirus 2 (Sars-CoV-2), despite our collective attempt and abilities to deal with the situation. We quickly understood the virus’ mechanism of transmission, identified and learned how to mitigate the most severe cases of the coronavirus disease 2019 (COVID-19), and prepared vaccines in record time. Unfortunately, ideological resistance, fueled by ignorance and the pursuit of profits, has undermined and continues to undermine this collective effort. Furthermore, clinical and epidemiological data show that COVID-19 has long-term effects that lead to post-COVID-19 neuropsychiatric disorders, including neurodegenerative diseases such as Alzheimer’s disease (AD; Heneka et al., 2020; Robinson-Agramonte et al., 2021; Toniolo et al., 2021).

In this review, we aim to discuss the neurochemical bases of these disorders, focusing on the role of Ca^2+^-binding proteins (CBP) associated with endoplasmic reticulum (ER) stress, particularly in astrocytes, which can result in an event known as an unfolded protein response (UPR). We address the steps and markers of this event. Then conclude by discussing drugs with potential neuroprotective actions against COVID-19, addressing two questions. First, we know that patients with diabetes mellitus are at increased risk of hospitalization and mortality for COVID-19 (Zhou et al., 2021). Could drugs already used in this disease, such as metformin, statins, and calcium channel blockers (CCB), provide some neuroprotection against COVID-19? Secondly, could these drugs affect the UPR and consequently contribute to reducing post-COVID-19 neurodegenerative diseases and/or cognitive impairment? These issues and questions will be discussed briefly in this commentary/review in an attempt to emphasize the role of reticular Ca^2+^ and CBP in brain activity and to broaden our understanding of the impact of COVID-19 on the development of neurodegenerative diseases.

## Astrocytes Are Integrative Players in Neuroinflammation

Cognitive decline features among the numerous and worrisome neurological and psychiatric manifestations associated with COVID-19. This manifestation results from many injurious factors, including direct viral aggression and systemic changes in the disease, such as coagulopathy, hypoxia, and cytokine storm, which all affect brain tissue, in addition to prolonged sedation in cases that require intubation and mechanical ventilation (Miners et al., 2020). However, to understand brain inflammation in COVID-19, whether by direct infection or due to ischemia or hypercytokinemia, we need to address the central regulatory role of astrocytes in the immune response (see Murta et al., 2020; Tremblay et al., 2020 for a review).

Astrocytes and microglia are the major cells that mount the inflammatory response in the central nervous system (CNS). However, unlike microglia, astrocytes are not immune cells, but when they receive molecular signs of injury they develop a complex machinery that makes them essential regulators of the adaptive and innate immune response (see Colombo and Farina, 2016; Sofroniew, 2020 for a review). Due to their heterogeneity and widespread location in the brain tissue, astrocytes facilitate communication with other cells as they coat the cerebral vascular endothelium, actively envelop the synapses (functioning as the third synaptic element), intimately contact the microglia and form a glial syncytial network through gap junctions, thereby functioning as integrating elements in the CNS. All this communication involves intense Ca^2+^ signaling from the ER (Verkhratsky and Parpura, 2014; Lia et al., 2021). The close functional relationship between neurons and astrocytes has supported the idea that astrocytic dysfunction accompanies or even precedes neuronal and cognitive damage in diseases such as diabetes mellitus and Alzheimer’s disease (González-Reyes et al., 2016).

Astrocytes respond to inflammatory cytokines, and produce cytokines in response to them and/or *via* the direct activation of receptors for damage-associated molecular patterns and pathogen-associated molecular patterns. In fact, they actively change under injury. For example, soluble tumor necrosis factor (sTNFα; molecular weight of 17 kDa) is recognized by receptor TNFR1 in astrocytes, which change and then start to express TNFR2, a typical receptor (but not exclusive to) on immune cells, which can recognize mTNFα (membrane-bound protein of 26 kDa) on visiting and neighboring cells (Fischer et al., 2014). These changes involve several transcription factors, such as nuclear factor kappa B (NF-kB), signal transducer and activator of transcription 3 (STAT3), and nuclear factor of activated T cells (NFAT), which in turn depend on Ca^2+^-mediated orchestration (Colombo and Farina, 2016; Schultz et al., 2021).

Significant infection and viral replication were observed in astrocytes of human brain cell cultures exposed to Sars-CoV-2, but minimal infection in other cell types was seen (Andrews et al., 2021). These data reinforce the astroglial commitment observed in brain postmortem studies of COVID-19 patients, where macrophage and T cell infiltration are observed, as well as vascular damage with fibrinogen leakage and extensive astrogliosis (Matschke et al., 2020). In fact, in postmortem studies, Sars-CoV-2 nucleocapsid was found in cortical astrocytes, as well as in neurons, microglia, and oligodendrocytes (Cama et al., 2021). Another very important aspect of COVID-19 is the greater vulnerability of elderly patients; possibly the aging of astrocytes makes these individuals more susceptible to Sars-CoV-2-induced dysfunction (Tremblay et al., 2020).

The plasma membrane enzyme angiotensin converting enzyme 2 (ACE-2), is involved in the anchoring and entry of Sars-CoV-2 in several cell types; however, in mature astrocytes, a role for this enzyme is debatable (Andrews et al., 2021). Nevertheless, the intracellular process of Sars-CoV-2 recognition apparently involves the activation of NOD-, LRR- and pyrin domain-containing protein 3 (NLRP3), a member of the pattern recognition receptors family (Heneka et al., 2020). NLRP3 has been associated with the development of degenerative diseases, such as diabetes mellitus (Volpe et al., 2016), and more recently associated with neurodegenerative diseases, possibly mediated by the UPR (discussed below; Guan and Han, 2020). This activation of NLRP3 by Sars-CoV-2 may support post-COVID-19 neurodegenerative diseases (Heneka et al., 2020).

In addition to the possibility of direct neuroinfection, the endothelial Sars-CoV-2-induced damage affects neural cells and can be triggered and/or aggravated by the ischemic inflammatory insult of COVID-19, which, in severe cases, progresses with atypical coagulation and hypoxia (Sashindranath and Nandurkar, 2021). In addition, hypercytokinemia (cytokine storm) in more severe cases of the disease can, through a mechanism involving astrocytes, mediate neurological damage, including cognitive deficit (Alnefeesi et al., 2021).

## UPR Steps and Markers

Currently, the understanding of neurodegenerative diseases involves changes associated with UPR, which result from ER stress and are often triggered by an imbalance in intracellular Ca^2+^ (van Vliet and Agostinis, 2016). We will review the UPR, emphasizing the main steps, CBP as markers of this event, and, finally, we will discuss a potential role for neuroviruses, in particular Sars-CoV-2, in the UPR in order to justify the observed increased risk of neurodegenerative diseases.

### UPR steps

The ER occupies a large structural and functional dimension in cell life. It possesses 60% of the membranous structure (in contrast to the plasma membrane, which has 5%) and is the major organelle responsible for the regulation of intracellular Ca^2+^ (Alberts et al., 2008). The ER is responsible for much of the synthesis, maturation, and folding of cellular proteins, including the transmembrane protein machinery involved in cellular secretion, emphasizing its importance for intercellular communication and the progression of diseases. Many physiological and pathological changes affect ER activity, such as ER-Ca^2+^ depletion, elevated protein synthesis and trafficking, glucose deprivation, inflammatory stimuli, hypoxia, and ROS, leading to ER stress (Bravo et al., 2013). Although the mechanisms are not all known, the changes mentioned are detected by ER stress sensors. There are three well-characterized transmembrane sensors; inositol requiring enzyme 1 (IRE1), protein kinase R-like ER kinase (PERK) and activating transcription factor 6 (ATF6). An additional sensor, functionally equivalent to ATF6, is found specifically in astrocytes, the old astrocyte specifically induced substance (OASIS); see Sims et al. (2022) for a review of these sensors in astrocytes. All these sensors, when activated, result in the restoration of reticular protein homeostasis (named proteostasis), i.e., they (a) reduce mRNA expression (e.g., *via* IRE1 activation) and protein synthesis (e.g., *via* PERK activation), contributing to lower protein traffic in the ER; (b) increase the synthesis of chaperones and other specific proteins that contribute to restoring the reticular function; and (c) increase the expression of proteins involved in the degradation of unfolded reticular proteins (*via* ATF6 activation), called ER-associated degradation (ERAD; Bahar et al., 2016; Martin-Jiménez et al., 2017).

**Figure 1:**
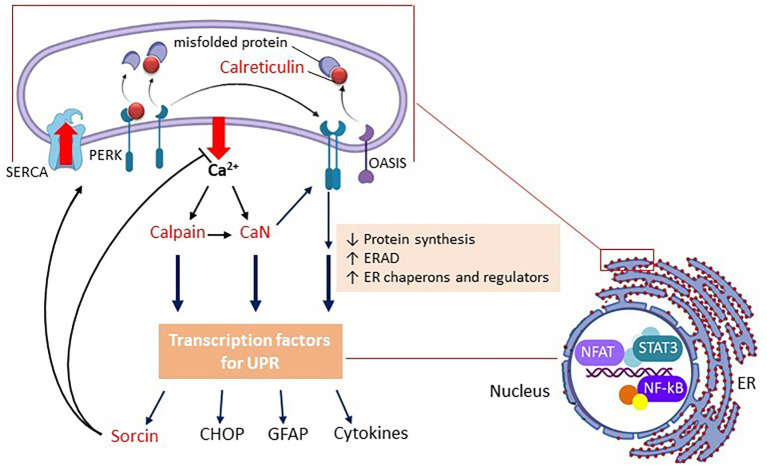
Schematic representation of the UPR in astrocytes. Only PERK and OASIS are represented as ER stress sensor proteins. Upon Ca^2+^ efflux, the chaperone CBPs (e.g., calreticulin) move from the sensor proteins (in the membrane) to the unfolded proteins (in the lumen), promoting the oligomerization of the sensor proteins, and triggering the UPR. In parallel, but not independently, the outgoing Ca^2+^ activates calpain and calcineurin (CaN). CBPs are all represented in red. Note that CaN (independent of enzyme activity) positively modulates PERK. Gene expression in the UPR, in general, is reduced, but some modulating proteins are upregulated, such as sorcin, CHOP, and GFAP, in addition to pro-inflammatory cytokines. Sorcin plays a role at the beginning of the UPR (negatively modulating Ca^2+^ efflux and CHOP expression). CHOP will play an important role in cell death if the triggering factor(s) of the UPR persist. It is important to mention that calpain and CaN modulate ER Ca^2+^-channels and SERCA, but in this scheme, only sorcin, which also plays this role, is highlighted. The image on the right emphasizes the structural relationship and bidirectional communication between the ER and the nucleus, where the main transcription factors of the astroglial inflammatory response (NF-kB, NFAT, and STAT) are highlighted. Abbreviations: CaN, calcineurin; CBP, calcium-binding protein; CHOP, CCAAT-enhancer-binding protein homologous protein; ERAD, ER-associated degradation; GFAP, glial fibrillary acidic protein; NF-kB, nuclear factor kappa B; NFAT, nuclear factor of activated T-cells; OASIS, old astrocyte specifically induced substance; PERK, protein kinase R-like ER kinase PERK; SERCA, sarco/endoplasmic reticulum Ca^2+^-ATPase; STAT, signal transducer and activator of transcription.

These sensor proteins are inactive, due to contact with Ca^2+^-binding chaperone proteins. Calreticulin (or glucose-regulated protein 94—GRP94) and immunoglobulin heavy chain-binding protein (BiP or GRP78) are the most abundant Ca^2+^-binding chaperones in the ER (Bravo et al., 2013). When these proteins move to fold the misfolded proteins in an attempt to restore the proteostasis compromised by ER stress, they activate the sensors. Therefore, the displacement of these chaperones (which are CBP) signals the ER stress and stimulates their further expression as a result of the activation of sensor proteins, thereby acting as primary markers of ER stress (see Figure 1).

### Extra-reticular UPR markers

In addition to the reticular chaperones, other proteins are synthesized to restore ER function. One of these, sorcin, has been shown more recently to play a multifunctional role in ER stress (Colotti et al., 2014). *So*luble *r*esistance-related *c*alcium-binding prote*in* (sorcin), like calpains, belongs to the penta-EF hand CBP family, which we will address later. Sorcin is a homodimer of 22 kDa that is highly expressed in the brain, heart, and several types of cancer cells, including glioma (Yokota et al., 2006). In fact, *resistance* in the name of this protein refers to its involvement in the resistance to chemotherapeutics in cancer cells. With regard to the UPR, this protein functions primarily by restoring reticular Ca^2+^ levels, inhibiting the Ca^2+^ efflux through ryanodine channels and stimulating entry through Ca^2+^-ATPase, as well as regulating L-type calcium channels and Na^+^-Ca^2+^ exchangers in the plasma membrane. Most data have been obtained in cardiomyocytes (Meyers et al., 1995; Matsumoto et al., 2005; Fowler et al., 2008) but the presence (Clemen et al., 2003) and co-localization of sorcin with ryanodine channels in astrocytes has been reported (Pickel et al., 1997). Taken together the data regarding brain sorcin indicate that this protein could be an early marker for neurodegenerative diseases (Genovese et al., 2020).

It should be pointed out that the UPR is an acute and adaptive response and, in situations in which triggering elements remain active, a new scenario arises, where UPR failure can lead to cell death, commonly mediated by the CCAAT-enhancer-binding protein homologous protein (CHOP; 29 kDa). Sorcin, in addition to regulating the flow of Ca^2+^, opposes ER stress, modulates the ATF6 sensor (Parks et al., 2021), likely promoting ERAD and inhibiting the synthesis of CHOP. However, all ER stress sensors potentially activate downstream signals for CHOP synthesis and prolonged reticular stress elevates CHOP, which in turn triggers cellular apoptosis involving several mechanisms (see Hu et al., 2019 for a review). These data emphasize the importance of the sorcin/CHOP ratio for characterizing the UPR phase and its association with degenerative diseases.

Calpains are also markers for the UPR. In fact, hypoxia and glucose deprivation of neurons cause UPR, associated with calpain elevation, which is able to induce apoptosis mediated by caspase activation (Wang et al., 2013; de la Cadena et al., 2014). In addition, exposure of APP/PS1 transgenic mice to hypoxia caused a calpain-mediated increment of beta amyloid deposition and tau phosphorylation (Wang et al., 2013). Calpains 1 and 2 are the best characterized calpains in brain tissue; they have a dimeric organization and weigh 80 kDa. The regulatory subunit is identical in calpains and both subunits have a domain containing penta-EF-hand calcium-binding motifs (Sorimachi and Ono, 2012), as observed in sorcin.

The role of calpains in astrocytes has been highlighted, particularly in neurodegenerative diseases such as AD (see Schultz et al., 2021 for a review). In fact, calpains are activated by reticular Ca^2+^, and many proteins involved in UPR that regulate the flow of this Ca^2+^ are targets of calpain (e.g., ryanodine channels). In addition to their direct role in Ca^2+^ signaling, these CBPs have many specific substrates that affect the cytoskeleton, gene expression, and protein degradation, which certainly reinforce their importance in UPR and cell fate.

Another key protein in UPR and a potential marker is calcineurin (CaN), which in turn is also a target of calpain (Mukherjee and Soto, 2011). CaN is the Ca^2+^-calmodulin protein phosphatase, which has numerous substrates and is a dimmer of 80 kDa, in which the catalytic subunit (CaN-A, 61 kDa) has two α and β isoforms in the brain tissue (Shah et al., 2017). This phosphatase is activated by the binding of calmodulin, a ubiquitous CBP, which displaces an auto-inhibitory domain, or by calpain, which cuts this domain (Wu et al., 2007). Other CBPs such as S100B (astrocyte-specific) and RCaN1 can also modulate CaN activity (Leal et al., 2004; Mitchell et al., 2007). Interestingly, induction of the UPR by Ca^2+^ depletion has been demonstrated in cultured neuronal cells, primarily due to an accumulation of gangliosides, caused a biphasic activation of the ER stress sensor PERK (Virgolini et al., 2019). An early (1 h) upregulation of CaN was observed and, under prolonged ER stress (48 h), an upregulation of CHOP was seen (where CaN expression was not different from controls), illustrating this mechanism discussed so far. This early increase in CaN was also observed in ER-Ca^2+^ depleted astrocytes in culture, where CaN (Aβ) was able to modulate PERK, promoting its oligomerization, independently of its phosphatase activity (Chen et al., 2016). This early protective effect of the astroglial CaN/PERK pathway was also identified in *in vivo* models of stroke and traumatic brain injury (Chen et al., 2016). This interaction of UPR-induced CaN (Aα) and PERK, as well as ER- Ca^2+^-ATPase, was previously described in cultured astrocytes (Bollo et al., 2010). Taken together, data indicate that CaN is a marker of UPR and has a protective role at the beginning of the response. However, prolonged astroglial CaN activation (involving calpain activity) may underly neuroinflammation and neurodegenerative diseases such as AD (Furman and Norris, 2014; Pleiss et al., 2016; Dos Santos et al., 2020).

It is not clear whether glial fibrillary acidic protein (GFAP), the classic marker of glial reactivity, can be measured to assess UPR in astrocytes. Data in the literature show that the increase in GFAP follows specific UPR markers, both in astrocyte cultures (Chen et al., 2016; Fan and He, 2016) and in *in vivo* injury models (Chen et al., 2016; Wang et al., 2019). GFAP has a complex gene regulation, where multiple transcription factors are potentially regulated by calpain and calcineurin. Apparently, all UPR sensors (Saito, 2014; Mehrbod et al., 2019) activation could affect GFAP gene expression.

### UPR spreading and viral infection

At this point, it is important to draw attention to a phenomenon that amplifies the impact of UPR, the reticular transmissibility in which molecules, during the UPR, can signal to neighboring cells, modifying the functionality of the ER, either preparing them for adaptation or inducing them to a functional failure. This phenomenon was first described in tumor cells (Mahadevan et al., 2011). Experiments using conditioned media from neural cell cultures (Meares et al., 2014; Sprenkle et al., 2017, 2019) emphasize the role of astrocytes in the transmissibility of ER stress. Surprisingly, although inflammatory mediators (cytokines) are the result of astroglial UPR, they are not the messengers of this transmissibility, which also does not seem to involve exosomes or vesicular particles.

**Figure 2:**
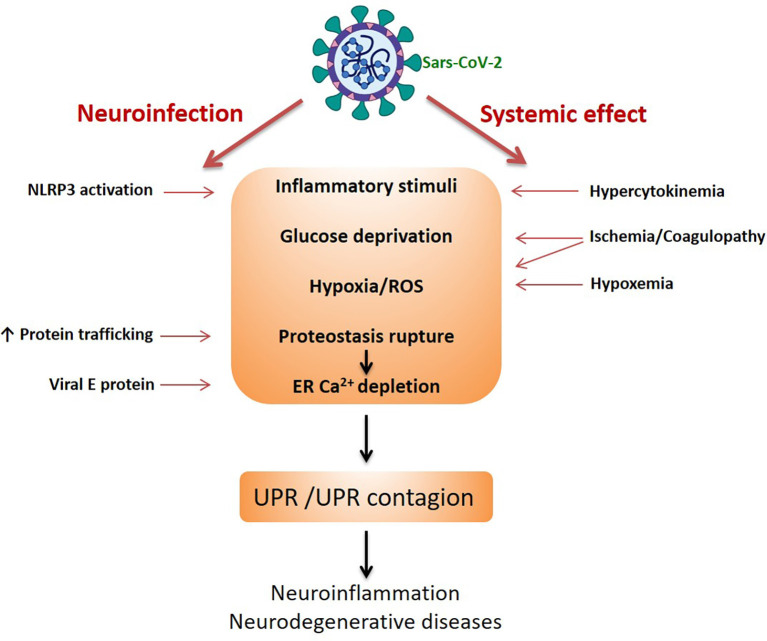
Sars-CoV-2-induced UPR in neural cells. The scheme highlights the two pathways of damage to the CNS by the coronavirus, indicating the possible mechanisms triggering UPR. The UPR and the intercellular spread of the UPR (UPR contagion) would be the basis of neurodegenerative diseases.

How can Sars-CoV-2 trigger UPR or contribute to its aggravation? Potentially, many viruses such as coronavirus, as well as arboviruses, influenza virus, and human immunodeficiency virus (HIV) are able to induce the UPR (Mehrbod et al., 2019). At the beginning of the pandemic, it was not known whether the virus could infect neural cells and whether the effects on the CNS observed were consequences of the systemic impact induced by the cytokine storm or by ischemia (due the coagulopathy, red blood cell agglutination and hypoxemia). We now know that Sars-CoV-2 affects the CNS through direct infection and through systemic effects (e.g., Murta et al., 2020). Figure 2 summarizes the routes and potential mechanisms that trigger UPR. In the case of Sars-CoV-2, it has been proposed that there is a huge demand for double membrane vesicles for viral replication (which come from ER) and increased protein traffic in the ER, due to the synthesis of structural transmembrane proteins of the virus, such as the S protein. This overloads ER activity and induces UPR (Prestes et al., 2021). Moreover, viral protein E is able to oligomerize and works as a viroporin, promoting Ca^2+^ efflux from the ER and consequently the UPR (Cao et al., 2021). In fact, confirming the occurrence of the UPR in coronavirus-infected cells, PERK activation and increases in GRP78 and GRP94 expression were detected (Chan et al., 2006; Palmeira et al., 2020). The commitment of astrocytes in patients with COVID-19 has been suggested by increases in blood serum GFAP, as well as neuronal damage from increased tau proteins and L neurofilaments (Pilotto et al., 2021; Virhammar et al., 2021). It is possible that the efflux of Ca^2+^, caused by protein E itself, could directly activate the NLRP3/inflammasome (Cao et al., 2021). Thus, reticular Ca^2+^ depletion leads to two possibilities; activation of the UPR (*via* the ER stress sensors) and/or inflammasome assembly, both of which are closely related mechanistically to the development of neurodegenerative diseases. Furthermore, it should be emphasized that, as mentioned, the UPR *per se* spreads, leading to what could be called a contagious UPR, without the need for the virus to be present.

## Putative Neuroprotective Drugs Against Post-COVID-19 Disorders

The first reports from COVID-19 showed a worse outcome in individuals with diabetes mellitus (Guo et al., 2020). These observations were accompanied by speculative attempts to explain the mediators involved, such as serum amyloid A protein (Gonçalves and Sesterheim, 2021) or arachidonic acid (Das, 2021). Furthermore, doubts persist as to whether medications used for the treatment of diabetes mellitus should be continued (Cheng et al., 2020; Fedson, 2020; Gao et al., 2020; Hariyanto and Kurniawan, 2020; Cariou et al., 2021; Kow et al., 2021). Some studies indicate benefits from the use of metformin, a blood glucose lowering agent, and statins, which inhibit the enzyme that regulates cholesterol synthesis. It is not our intention to discuss all the possible mechanisms of these drugs but to seek their associations with the UPR and, consequently, with their potential to attenuate or prevent the development of neurodegenerative diseases, associated with cognitive deficit and dementia. It should be noted that all of these drugs act beneficially on astrocytic functions in different models of injury; however, their specific role in the UPR of these cells is less explored (e.g., Déry and LeBlanc, 2017; Natrus et al., 2022). A summary of the data presented in this section about the effects of metformin, statins, and Ca^2+^ channel blockers (CCB) on UPR and neuroprotection is presented in Table 1.

**Table 1:** Potential neuroprotective effect of drugs prescribed to diabetic patients.

**Drug/Effect on**	**UPR^a^**	**Covid-19 outcome^b^**	**Cognitive impairment^c^**	**Covid-19/ Neuroprotection^d^**	**References**
Metformin	↓	Favorable	Only for moderate cognitive impairment	Yes, based on experimental data; maybe, based on clinical studies	Luchsinger et al. (2016), Ibrahim et al. (2021), Zangiabadian et al. (2021), and Kastora et al. (2022)
Statins	↓↑	Favorable in non-severe patients	Cognitive benefits, but do not modify the risk for dementia	Yes, based on experimental data	McGuinness et al. (2016), Chow et al. (2021), and Vahedian-Azimi et al. (2021)
CCB^e^	↓	Favorable, based on some reports	May improve cognition	Yes, based on experimental data	Lawlor et al. (2018), Zhang et al. (2020), Crespi and Alcock (2021), and Cunningham et al. (2021)

^a^See Figure 3 for details about molecular mechanisms (↓decrease or ↑increase UPR); ^b^risk of hospitalization and mortality in diabetic patients with Covid-19; ^c^long-term use in diabetes mellitus patients; ^d^possible protection against the neural aggression of Sars-CoV-2; ^e^Ca^2+^-channel blockers.

**Figure 3:**
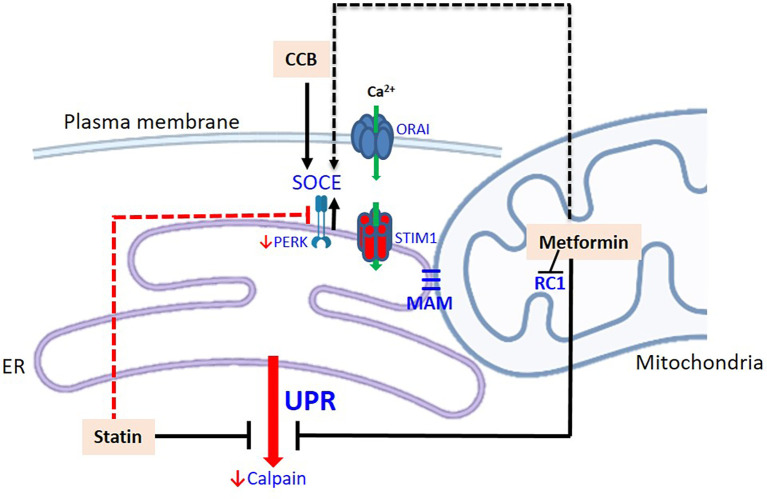
Modulation of the UPR by drugs prescribed for patients with diabetes mellitus. Metformin inhibits the respiratory chain complex 1 (decreasing ROS) and consequently the efflux of Ca^2+^ from the ER. This could potentially stimulate Ca^2+^ entry *via* SOCE. Statins down-regulate the expression of CBP chaperones of the ER, calpains, and PERK. Potentially, statins have a dual role, as downregulation of PERK can decrease Ca^2+^ entry into the ER, *via* SOCE. CCB stimulates STIM-1 and increase the input of Ca^2+^ to the ER. The dashed routes refer to potential pathways and their effects on neural cells, particularly astrocytes, need to be confirmed. Abbreviations: CCB, calcium channel blockers; MAM, mitochondria-associated ER membrane; RC1, respiratory chain complex 1; STIM-1, stromal-interacting molecule 1.

People with diabetes mellitus (with poor glycemic control) are more prone to hospitalization and worse outcome following COVID-19 infection (Singh et al., 2020; Schlesinger et al., 2021). Since metformin is a blood glucose reducer, the benefit of this drug seems obvious. However, insulin does not provide the same protection in COVID-19 patients, suggesting that the effect does not depend exclusively on lowering blood glucose (Kastora et al., 2022). The effect of metformin is attributed to the activation of AMP-activated protein kinase (AMPK), a key enzyme in cell metabolism (see Varghese et al., 2021 for a detailed review). However, the primary action of metformin is the inhibition of mitochondrial complex 1, which alters the AMP/ATP ratio. This inhibition, in addition to increasing the levels of AMP, reduces the generation of mitochondrial ROS, which in turn, due to the structural and functional intimacy between the organelles, through the MAMs (mitochondria-associated endoplasmic reticulum membranes; Vance, 2014), reduces the efflux of reticular Ca^2+^ (Figure 3). Furthermore, metformin appears to inhibit store-operated calcium entry (SOCE; Soberanes et al., 2019), which we will address later.

The ability of metformin to reduce the UPR (based on GRP94 and CHOP changes) was initially reported in renal tubular cell cultures, where the effect was independent of AMPK activation (Thériault et al., 2011). More recently, the abrogating effect of metformin against the UPR and its neuroprotection has been confirmed in STZ-induced diabetic mice (Docrat et al., 2020) and senescence-accelerated mice (Liu et al., 2020). Moreover, the protection of neural cells by metformin could involve the inhibition of Cdk5 activation by inhibiting calpain, a downstream UPR enzyme (Wang Y. et al., 2020). These findings indicate possible neurological benefits when maintaining metformin use in diabetic patients during COVID-19, although neuroprotection has not been properly evaluated (Ibrahim et al., 2021; Zangiabadian et al., 2021). In fact, benefits were observed in patients with a moderate cognitive deficit, who were properly assessed with the ADAS-Cog (AD assessment scale—cognitive subscale; Luchsinger et al., 2016). However, the use of metformin and its neuroprotective potential cannot be seen as a panacea for post-COVID-19 neurological disorders, as other pre-COVID-19 results contradict this neuroprotection (Ping et al., 2020).

Statins are cholesterol-lowering compounds and are the first drug of choice for the treatment of dyslipidemias in cardiovascular disease and diabetes mellitus patients (van Stee et al., 2018). Although the mechanism of action of statins involves the reduction of cholesterol synthesis by inhibiting the enzyme, HMG-CoA reductase, the effects of these compounds are potentially very wide, as they not only affect the synthesis of cholesterol and derivatives but also affect the prenylation of many proteins. This justifies, in part, the pleiotropic effect of statins, including their anti-inflammatory and neuroprotective effects (Barone et al., 2014; Oesterle and Liao, 2019). Clinical benefits of statins in COVID-19 patients were raised based on their anti-inflammatory effects, particularly on NF-kB and NLRP3 (e.g., Liu et al., 2021). In fact, a recent meta-analysis suggests that in-hospital statin use leads to a significant reduction in all-cause mortality in COVID-19 cases (Vahedian-Azimi et al., 2021). However, some results are questionable, indicating clinical benefits only in non-severe COVID-19 patients (Fedson, 2020; Chow et al., 2021; Hariyanto and Kurniawan, 2021).

The use of statins is currently widespread with the expectation of a reduction in cognitive deficits with age and a reduction in the risk of dementia (Schultz et al., 2018). Clinical trials with statins in cardiovascular disease (CVD) patients indicate benefits, but suggest that they do not modify the risk for dementia (Gurm and Hoogwerf, 2003; McGuinness et al., 2016). However, experimental data support this possibility since statins reduce UPR markers in different models of ER stress in brain tissue (Urban et al., 2009; Liu et al., 2018; Mounier et al., 2021), although the mechanism remains unknown (Mollazadeh et al., 2018). Calpains could be targets of statins, since their expressions are downregulated in neural and non-neural cells (Li et al., 2015; Han et al., 2019).

Interestingly, some calpain inhibitors have been proposed as antiviral compounds against Sars-CoV-2 infection (Rut et al., 2020; Sacco et al., 2020; Milligan et al., 2021). The viral target of calpain inhibitors is the main protease (Mpro or Nsp5), responsible for activating/release of many non-structural viral proteins (Nsp), such as the RNA-dependent RNA polymerase (Nsp12); inhibition of Mpro prevents viral replication. However, caution is needed with these potential antiviral compounds as they affect a key enzyme for host UPR, which in turn affects multiple substrates in various cellular activities (Sorimachi and Ono, 2012). Calpain inhibitors for AD are also in preclinical studies (Lon et al., 2019) and these investigations should take into account the cell-specific effects of calpain. Calpain appears to favor beta amyloid formation and accumulation in neurons, but it is possible that it is responsible for the beta amyloid clearance performed by astrocytes (Schultz et al., 2021).

In addition to calpains, the expressions of other UPR proteins are also downregulated by statins, such as GRP78 and GRP94, and the sensor protein PERK itself (Mollazadeh et al., 2018). Therefore, statins could reduce the UPR as a whole. It is noteworthy that PERK can play a modulating role in store-operated calcium entry (van Vliet and Agostinis, 2016). SOCE is the main reticular Ca^2+^ replacement mechanism. ER Ca^2+^ depletion is detected by the CBP chaperones, which detach from the ER stress sensor proteins, as already discussed (see Figure 1), and are detected by the transmembrane reticular protein, STIM1 (stromal-interacting molecule 1; Figure 2), which (like the ER stress sensor proteins) oligomerizes. STIM1 has its own EF-hand calcium-binding sites that “feel” Ca^2+^ depletion. Oligomeric STIMs connect to specific plasma membrane Ca^2+^ channels and allow Ca^2+^ to enter to replenish reticular Ca^2+^, working in coordination with ER Ca^2+^ ATPase. Activated PERK could modulate the connection of STIM with membrane channels, promoting Ca^2+^ replenishment (van Vliet and Agostinis, 2016).

Thus, statins may potentially have a dual role, as they are able to reduce the activation of the UPR, and also reduce the replenishment of reticular Ca^2+^ (by decreasing PERK expression). This duality may contribute to understanding the antitumor role of statins. In fact, inhibition of PERK by simvastatin favors the death of U87 glioblastoma cells (Dastghaib et al., 2020). The final result would depend on the machinery involved, varying in each cell type. Cultured astrocytes express reticular STIM1 and the plasma membrane Ca^2+^ channels, ORAI/CRAC 1–3, and TRPC1, which when coupled to STIM1 mediate the replacement of Ca^2+^ (Kwon et al., 2017).

Other potential modulators of the UPR, Ca^2+^ channel blockers (CCB), have also been widely used to treat hypertension in CVD and diabetes mellitus patients (Bergantin, 2019; Zhu et al., 2021). These drugs block L-type voltage-dependent Ca^2+^ channels (LVCC), reducing cardiac and vascular contractility. Several reports show the benefits of these drugs in COVID-19, where they reduce the need for intensive care and mortality (Zhang et al., 2020; Crespi and Alcock, 2021). Experimental data have reinforced the possible benefits of these drugs in COVID-19 cases (Manohar et al., 2021; Straus et al., 2021).

As we mentioned, SOCE is closely related to ER stress and, consequently, to neurodegenerative diseases (van Vliet and Agostinis, 2016; Secondo et al., 2018). In addition to these channels, it is well documented that blocking LVCC can also prevent ER stress (e.g., Wang et al., 2011). Although the mechanism is not completely understood, a positive modulation of STIM-1 by CCB in vascular smooth muscle cells has been suggested (Johnson et al., 2020). In agreement with the possibility of neuroprotection, the induction of hippocampal UPR by the infusion of beta amyloid peptides into the entorhinal cortex was blocked by CCB, which not only reversed the increases in markers (GRP78 and CHOP) but also improved the cognitive deficit in these animals (Ghanbari-Maman et al., 2019). In other words, these blockers may represent potential neuroprotective drugs in COVID-19, but broader clinical studies such as those performed with ACE inhibitors for the treatment of hypertension in COVID-19 patients, are still lacking (Xu et al., 2021). It is worth remembering that clinical studies did not convincingly show that antihypertensive drugs (including CCB) could reduce cognitive impairment or the risk of dementia (Cunningham et al., 2021). However, a trial with CBB nilvadipine was able to improve cognition (assessed by the ADAS-Cog) in a population with a moderate cognitive deficit (Lawlor et al., 2018).

It is important to raise some criticisms of the pharmacological therapeutic approach of this review and of how we have conducted this discussion. Metformin, statins and CCB potentially act by blocking UPR or activating SOCE as possible protective mechanisms. Parenthetically, recent results in cardiomyocytes indicate that metformin, in addition to blocking the UPR, could potentially stimulate SOCE, where it acts by blocking L-type channels (Wang H. et al., 2020; see Figure 3). However, we cannot take such a simplified view of intervention. In AD, a reduction of STIM-1 has been reported and, therefore inhibiting SOCE could aggravate the disease. Conversely, inhibition of SOCE seems to be neuroprotective in Huntington’s disease (Secondo et al., 2018). Similarly, SOCE blockade provided neuroprotection to cultured neurons submitted to hyperglycemia (Xu et al., 2016). Another aspect that deserves attention is the fact that neural cell heterogeneity is commonly reduced to the neuron. We often discuss neuroprotection as neuronal protection, and do not value the dysfunction of other cells, in particular astrocytes, which may precede neuronal dysfunction. Finally, it is worth emphasizing that we often discuss UPR and SOCE without taking into account the nature of the stimulus that is inducing the response; this can lead to undue generalizations. For example, cultured astrocytes in the presence of LPS respond with a decrease in SOCE, but respond to amyloid β42 with an increase in SOCE (Ronco et al., 2014).

## Concluding Remarks

We would like to summarize some ideas, based on references presented throughout the text. A large quantity of material has been, and is still being, produced about the Sars-CoV-2 pandemic. We have addressed a part of these studies and, therefore, such ideas are possibly far from the conclusions that we will learn from this pandemic with regard to its impact on the CNS, especially in diabetic individuals. However, these ideas express, with many limitations, the result of our attempt to establish a link between UPR (aggravated by Sars-CoV-2) in neural cells and possible neurological outcomes. (i) We have advanced in understanding the efflux and influx of Ca^2+^ in the ER, which are structurally and functionally coupled to the plasma membrane and mitochondria. Depletion of reticular Ca^2+^ induces displacement of CBP chaperones and activation of sensor proteins, initiating the UPR. The long-lasting UPR is the trigger for many neurodegenerative diseases. (ii) Considering the role of the astrocyte network in neuronal communication, in immune defense and in the metabolic integration of the brain, we can suggest that the astroglial UPR (and the possibility of “reticular contagion” between cells) may trigger neuroinflammation and neurodegenerative diseases, and represent a target for therapeutic strategies. (iii) As astrocytes are targets of Sars-CoV-2 (and other infectious agents) we may predict the impact of the virus on the activity of brain cells in COVID-19 and post-COVID-19, which may aggravate the ongoing UPR or trigger neurodegenerative diseases. (iv) Our knowledge and therapeutic arsenal (metformin, statins, and CCB) against the UPR, although useful, is still very limited. The pandemic has put us on the spot, but now we have to advance further in understanding these and other neuroprotective drugs, going beyond assessing clinical severity and mortality. (v) We have to advance in the translational investigation of neuroprotection, with more detailed clinical assessment tools to evaluate cognition. All projections about neurodegenerative diseases, and in particular AD, that we made before the pandemic, taking into account the increase in life expectancy and the increase in non-communicable diseases (such as diabetes mellitus), may have aged.

## Author Contributions

All authors contributed to the article (conception and elaboration) and approved the submitted version.

## Funding

This research was supported by Conselho Nacional de Desenvolvimento Científico e Tecnológico (CNPq), Coordenação de Aperfeiçoamento de Pessoal de Nível Superior (CAPES), Fundação de Amparo à Pesquisa do Estado do Rio Grande do Sul (FAPERGS, PPSUS 21/2551-0000067-8) and Instituto Nacional de Ciência e Tecnologia para Excitotoxicidade e Neuroproteção (INCTEN 465671/2014-4).
